# Supplementary figures and images for: Recognition and differentiation of dural puncture click sensation: A subjective and objective prospective study of dural puncture forces using fine-gauge spinal needles
Source: PLoS One. 2021 Jul 30;16(7):e0247346. doi: 10.1371/journal.pone.0247346 (PMC8323951; doi:10.1371/journal.pone.0247346)

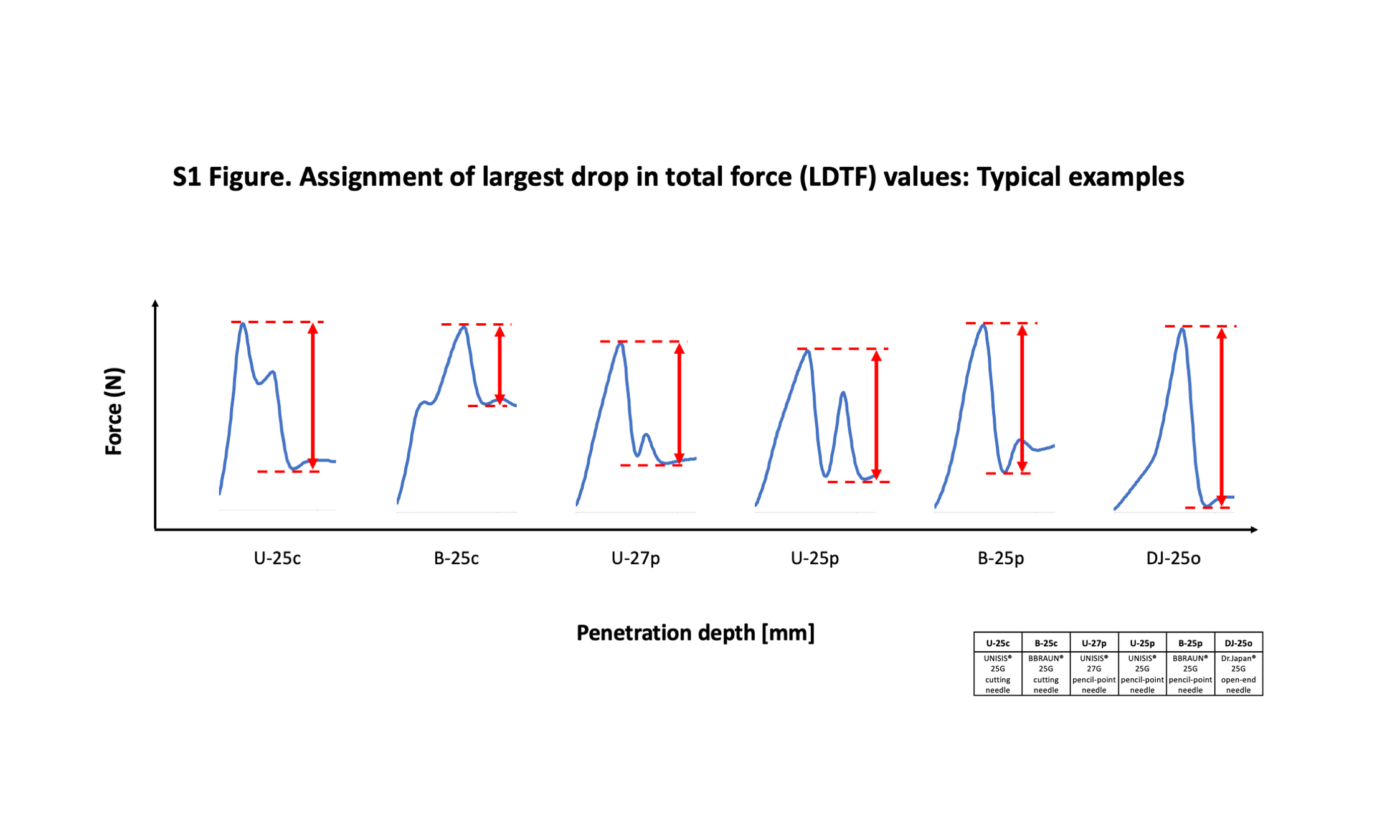

Supplement: S1 Fig — The largest drop in total force (LDTF) between the local maximum and minimum on the load-displacement curve for each needle was defined to reflect the subjective click perception expressed in numerical rating scale values. The local maximum was the peak total force value in the load-displacement curve data, while the local minimum value was the nearest lowest total force value observed in the data set after rapid drop(s) in force when the difference with the peak total force value was the largest. (TIF) [file pone.0247346.s001.tif]
